# Maternal Serum VEGF Predicts Abnormally Invasive Placenta Better than NT-proBNP: a Multicenter Case-Control Study

**DOI:** 10.1007/s43032-020-00319-y

**Published:** 2020-10-06

**Authors:** Alexander Schwickert, Frédéric Chantraine, Loreen Ehrlich, Wolfgang Henrich, Mustafa Zelal Muallem, Andreas Nonnenmacher, Philippe Petit, Katharina Weizsäcker, Thorsten Braun

**Affiliations:** 1 Department of Obstetrics, Charité – Universitätsmedizin Berlin, Corporate Member of Freie Universität Berlin, Humboldt-Universität zu Berlin, and Berlin Institute of Health, Augustenburger Platz 1, 13353 Berlin, Germany; 2 Department of Obstetrics and Gynecology, Centre Hospitalier Universitaire de Liège, site CHR Citadelle, Blv 12ème de Ligne 1, 4000 Liège, Belgium; 3 Department of Gynecology with Center for Oncological Surgery, Charité – Universitätsmedizin Berlin, Corporate Member of Freie Universität Berlin, Humboldt-Universität zu Berlin, and Berlin Institute of Health, Augustenburger Platz 1, 13353 Berlin, Germany; 4 Department of Experimental Obstetrics, Charité – Universitätsmedizin Berlin, Corporate Member of Freie Universität Berlin, Humboldt-Universität zu Berlin, and Berlin Institute of Health, Augustenburger Platz 1, 13353 Berlin, Germany

**Keywords:** Vascular endothelial growth factor, Placenta increta, Placenta percreta, Abnormally invasive placenta, Placenta accreta spectrum, Biomarker

## Abstract

The aim of this study was to test if maternal serum vascular endothelial growth factor (VEGF) or N-terminal pro B-type natriuretic peptide (NT-proBNP) predicts abnormally invasive placenta (AIP) better. Secondary objective was to test whether the serum levels of VEGF and NT-proBNP can predict the degree of invasion. In a multicenter case–control study design, gestational age-matched serum samples from pregnant women with AIP (*n* = 44) and uncomplicated pregnancies (*n* = 55) who had been enrolled at Charité – Universitätsmedizin Berlin, Germany and Centre Hospitalier Régional de la Citadelle in Liège, Belgium were analyzed. Maternal blood serum VEGF and NT-proBNP levels were immunoassayed from samples taken immediately before delivery (GA median: 35 weeks). Biomarker levels were compared between AIP and control group. The correlation of biomarker levels with the clinical AIP degree was assessed. The predictive biomarker ability was characterized through a multivariate regression model and receiver operating characteristic curves. Women with AIP had significantly lower maternal serum VEGF levels (AIP mean 285 pg/ml, 95% CI 248–322, vs. control: 391 pg/ml, 95% CI 356–426, *p* < 0.01) and higher NT-proBNP levels (AIP median 329 pg/ml, IQR 287–385, vs. control 295 pg/ml, IQR 273–356, *p* = 0.03). Maternal serum VEGF levels were able to predict AIP better (AUC = 0.729, 0.622–0.836, *p* < 0.001; VEGF + number of previous cesarean deliveries: AUC = 0.915, 0.853–0.977, *p* < 0.001). Maternal serum VEGF levels correlated inversely with the clinical AIP degree (*r* = − 0.32, *p* < 0.01). In short, maternal serum VEGF, more than NT-proBNP, can help in predicting AIP and hints at the degree of invasion.

## Introduction

Abnormally invasive placenta (AIP) describes the clinical situation where a placenta does not separate spontaneously at delivery and cannot be removed without causing abnormal and potentially life-threatening bleeding [1, 2].

Safe delivery in pregnancies complicated by AIP depends on the timely referral of a pregnant woman to a center specialized in treating placental pathologies, especially in the case of placenta percreta (AIP degree of invasion 3A to 3C according to FIGO classification) [3]. Ultrasound as the gold standard for diagnosing AIP in specialized hands yields a sensitivity and specificity of over 90% [4–7]. However, recent population studies from the UK, the USA, and northern Europe have shown that AIP remains undiagnosed before delivery in between half to two thirds of cases [8–10]. Reasons include limited availability of specialized ultrasound scans that are able to detect the frequently subtle ultrasound features of AIP entities such as invasion of the lower posterior bladder wall or the parametria [11–13]. Therefore, suitable maternal serum biomarkers might aid doctors additionally to ultrasound in gynecological practices worldwide in detecting AIP early and for planning the appropriate management for pregnant women with AIP [14–16].

Vascular endothelial growth factor (VEGF) and N-terminal pro B-type natriuretic peptide (NT-proBNP) are known to play a role in the process of placentation [17–20]. Both show altered serum levels in AIP cases, having potential as promising biomarker candidates [21, 22]. However, there has been no evaluation of these candidates against the background of different degrees of invasion of AIP, and their ability to predict AIP has yet to be comprehensively compared. VEGF promotes angiogenesis, chemotaxis, and vasodilation. It is expressed by the amniotic epithelium and the cytotrophoblast [17, 18]. In women with AIP (Wehrum et al.: *n* = 13, Uyanikoglu et al.: *n* = 22), maternal serum VEGF levels during third trimester are significantly lower compared with women with normal placentation (0.8 (0.02–3.4) vs 6.5 (2.7–10.5) pg/mL, *p* = 0.02; 39.18 ± 11.98 vs 85.87 ± 18.05 pg/ml, *p* < 0.001) [21, 23]. However, Biberoglu et al. did not find significantly differing serum levels for VEGF in AIP cases compared with normal placentation (*n* = 65) [24]. NT-proBNP has recently been shown to enhance vasculogenesis [20]. As women with early- (365 (14–9815) pg/ml) and late-onset preeclampsia (176 (33–2547) pg/ml) have higher plasma levels compared with controls of similar gestational age (*p* < 0.001), a placental production of NT-proBNP has been hypothesized [19]. Women with AIP seem to exhibit higher serum NT-proBNP levels than women with normal placentation [22]. It therefore appears plausible that NT-proBNP plays a role in placental vasculogenesis and development.

The aim of this study was to compare maternal serum VEGF and NT-proBNP-levels in women with different degrees of AIP and a high share of women with placenta percreta and to test whether these biomarker levels can predict the clinical degree of invasion.

## Methods

### Study Design and Patient Population

In a case–control study design, maternal blood serum samples were taken immediately before delivery from 99 women with singleton pregnancies enrolled prospectively at Charité – Universitätsklinikum in Berlin, Germany, and Centre Hospitalier Régional de la Citadelle in Liège, Belgium. The study group consisted of 44 patients (*n* = 17 in Liège, *n* = 27 in Berlin; gestational age [GA] median 35 weeks; interquartile range (IQR) 35–36 weeks) who had been diagnosed with AIP antenatally. Blood specimens taken immediately before delivery from 55 healthy women pregnant with singletons, who had been previously recruited at the Charité – Universitätsklinikum during another trial, were matched for GA (median 35 weeks; IQR: 33–37 weeks) retrospectively serving as controls [25–27]. Women with conditions that might influence serum VEGF and NT-proBNP levels such as cancer, preeclampsia, hypertension, or heart failure were not included in the study. All women had provided signed informed consent for blood retrieval and serum analyses under protocols approved by the Ethics Committees of Charité – Universitätsklinikum Berlin (EA1_031_15, EA2_149_07) and Centre Hospitalier Régional de la Citadelle in Liège (B412201319082).

### Timing of Delivery

Planning of the delivery was done according to FIGO consensus guidelines and the evidence-based guidelines for the management of AIP which recommend to “continue expectant management until after 36+0 weeks’ gestation for women with no previous history of preterm delivery (<36+0 weeks) and who are stable with no vaginal bleeding, preterm premature rupture of membranes, or uterine contractions suggestive of pre-term labor” [2, 28].

### Ultrasonographic Evaluation and Diagnosis of Abnormally Invasive Placenta

The prenatal diagnosis of AIP was made through gray-scale, pulsed wave and color Doppler ultrasound imaging based on the previously published standardized ultrasound descriptors of AIP [29]. Most women were referred after suspicion of AIP during second trimester ultrasound scans. Women who did not have a detailed second trimester ultrasound scan were diagnosed upon their first visit to the clinic for counseling about birth mode after previous cesarean section or upon presentation in the emergency room. Grading of AIP was done intraoperatively according to the classification adopted by the FIGO from grades 1 to 3C [3]. In short, grade 1 signifies placenta accreta, grade 2 signifies placenta increta, grade 3A signifies placenta percreta limited to the uterine serosa, grade 3B signifies placenta percreta with urinary bladder invasion, and grade 3C signifies placenta percreta with invasion of other pelvic tissue/organs [3].

### Blood Collection, Storage, and Immunoassay Procedures

All maternal blood samples were retrieved by venipuncture. To avoid surgery- or birth-associated affection of the biomarker levels, blood samples were taken preoperatively in cases of planned cesarean deliveries or at admission to the hospital in cases of attempted vaginal birth. Serum samples were spun at 3.500×*g* at room temperature for 10 min; the supernatant was aliquoted and immediately stored at −80 °C until VEGF and NT-proBNP levels were measured using specific immunoassays [24]. Enzyme-linked immunosorbent assays for human unbound VEGF-A and NT-proBNP were performed according to the manufacturer’s instructions (VEGF-A: Product No. SEA143Hu, Cloud-Clone Corp., Katy, USA; NT-proBNP: Catalog No. E01B0203, BlueGene, Shanghai, China) with an immunoassay device (FLUOstar Omega, BMG Labtech, Ortenberg, Germany) by investigators blinded to the patients’ group classification. Both assays were run with positive controls and validated with the inter-assay coefficient of variability (VEGF: 4.5%, NT-proBNP: 8.5%).

### Statistical Analysis

Data were tested for normality assessing the histogram and using the Kolmogorov–Smirnov test. Comparisons between groups were performed using Student’s *t* test for independent samples and Mann–Whitney *U* test. Categorical variables were expressed as numbers (percentage) and compared using chi-square test. Data are reported as median and interquartile range (IQR) or mean and 95% confidence interval (95% CI), as appropriate. Sample size calculation for the primary outcome of AIP vs. normal placentation was based on the publications by Uyanikoglu and Ersoy (*α* = 0.05%, power = 90%, *t* test/ANOVA) [22, 23]. It was estimated that at least 6 patients per group would be necessary to detect differences between five groups and at least 31 per group for two groups. Correlations between variables were explored using Spearman’s rank order correlations for non-normally distributed continuous variables. A multivariate regression model was used to indicate predictors of AIP. In a first step, crude odds ratios with 95% confidence interval (95% CI) were calculated for each variable separately (univariate analysis). In a second step, adjusted odds ratios were calculated with multivariate regression, including variables that had been identified as having a significant effect in univariate analysis (i.e., *p* < 0.05). The true-positive and false-positive rates for the diagnosis of AIP were estimated from receiver-operating characteristic (ROC) curves, and the diagnostic powers of the biomarker levels were assessed from the respective area under the curve (AUC). Cut-off values for maternal serum VEGF and NT-proBNP levels were calculated using Youden’s index [30, 31]. SPSS Version 25 (IBM, Chicago, USA) was used for statistical analysis. Figures were prepared with Prism 8 (GraphPad Software, San Diego, USA). In all analyses, two-tailed *p* ≤ 0.05 were considered to indicate statistical significance.

## Results

### Clinical Characteristics of Women and Management of AIP

Demographic, clinical, and pregnancy outcome characteristics of the cohort are presented in Table 1. As expected, women with AIP were significantly older (34 (26–38) vs. 30 (32–37) years, *p* < 0.001), of higher gravidity (4 (3–5) vs. 2 (2–4), *p* < 0.001) and parity (2 (1–3) vs. 1 (1–2), *p* < 0.001) and had a higher number of prior cesarean deliveries when compared with controls (2 (1–3) vs. 0 (0), *p* < 0.001) (Table 1). Placenta previa was diagnosed in 86% of women with AIP and in 9% of pregnancies with normal placentation. Mean gestational age at diagnosis in the two study centers based on ultrasound findings was 27 weeks (95% CI 25–29 weeks). There was no significant difference between the two centers in Belgium and Germany (28 (26–31) vs. 26 (24–29) weeks at diagnosis, *p* = 0.34). In an intensive pre-operative counseling, the management of AIP was discussed with the patient and primarily based on the wish and in consent with the patient. Four women preferred a conservative approach with leaving the placenta in situ. Three of them experienced successful placental resorption without further procedures. One had to undergo delayed hysterectomy 3 months after cesarean section. Focal resection was performed in women who wished to conserve their uterus provided that sufficient myometrium for an optimum closure of the uterus was available after the resection. Overall, 73% of women with AIP were clinically classified as AIP degree 3A to 3C (i.e., placenta percreta) and histopathologically confirmed. In the AIP group, 30 women (68%) received a hysterectomy (3 (43%) in AIP Grade 2, 3 (75%) in AIP Grade 3, 17 (74%) in AIP Grade 4, 5 (63%) in AIP Grade 5, 2 (100%) in AIP Grade 6). Statistically significant *p*-values (<0.05) are written in italics.

**Table 1:** Demographic, clinical, and outcome characteristics of enrolled women (*n* = 99)

Variable	AIP (Grades 1–3C) (*n* = 44)	Controls (*n* = 55)	*P* value
Characteristics at enrolment			
Maternal age (year)^a^	34 (26–38)	30 (32–37)	*< 0.001*
BMI^a^	29 (27–30)	28 (26–29)	0.29
Gravidity^b^	4 (3–5)	2 (2–4)	*< 0.001*
Parity^b^	1 (1–2)	2 (1–3)	*< 0.001*
Number of prior D&C^c^			0.711
0 (no prior D&C)	27 (61)	34 (62)	
1 prior D&C	11 (25)	11 (20)	
≥ 2 prior D&C	6 (14)	10 (18)	
Number of prior cesarean deliveries^c^			*< 0.001*
0 (no prior cesareans)	4 (9)	43 (78)	
1 prior cesarean	17 (39)	9 (16)	
≥ 2 prior cesareans	23 (52)	3 (6)	
Placenta previa^c^	38 (86)	5 (9)	*< 0.001*
Outcome characteristics			
Gestational age at delivery in weeks^b^	35 (35–36)	35 (33–37)	0.397
Cesarean delivery^c^	44 (100)	18 (33)	*< 0.001*
Cesarean hysterectomy^c^	30 (68)	0	*< 0.001*
Focal resection^c^	7 (16)	0	*< 0.001*
Leaving placenta in situ^c^	4 (9)	0	*< 0.001*
Manual removal of placenta^c^	4 (9)	0	*< 0.001*
AIP grading^c^			*< 0.001*
Normal placentation	0	55 (100)	
1	6 (13.6)	0	
2	6 (13.6)	0	
3A	22 (50)	0	
3B	7 (16)	0	
3C	3 (6.8)	0	
Blood loss in milliliter^a^	1865 (1464–2266)	367 (305–430)	*< 0.001*
Blood transfusion^c^			*< 0.001*
0 red packed cells	22 (51)	55 (100)	
≤ 4 red packed cells	15 (35)	0	
≥ 5 red packed cells	6 (14)	0	

*Abbreviations*: *AIP* abnormally invasive placenta, *D&C* dilation and curettage

^a^Data presented as mean (95% CI) and analyzed by *t* test

^b^Data presented as median (IQR) and analyzed by Mann–Whitney *U* test

^c^Data presented as *n* (%) and analyzed by chi-squared test

### Circulating Levels of VEGF and NT-proBNP and the Occurrence of Abnormally Invasive Placenta

**Fig. 1:**
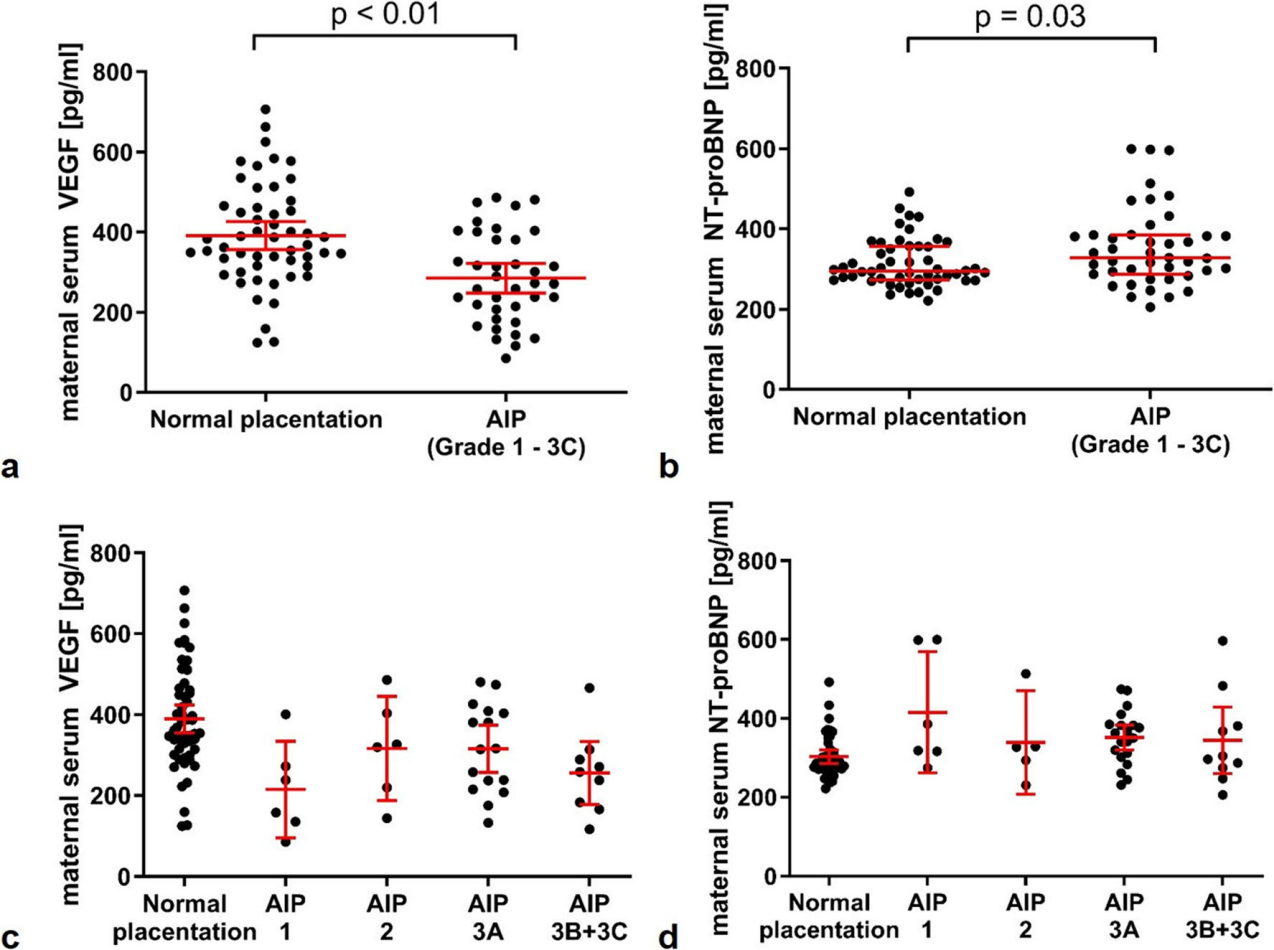
Angiogenic factors in women with abnormally invasive placenta. Scatter plot showing the significant **a** decrease of maternal serum VEGF and **b** increase of maternal serum NT-proBNP in women with abnormally invasive placenta (VEGF: *n* = 38, NT-proBNP: *n* = 43) as compared with gestational age-matched controls (*n* = 54). Values are shown as mean ± 95% confidence interval (95% CI) for VEGF and median ± interquartile range (IQR) for NT-proBNP. The median of gestational age at delivery was 35 weeks in both groups (AIP: IQR 35–36; Controls: IQR 33–37). **c, d** VEGF and NT-proBNP levels according to AIP degree. Scatter plot showing maternal serum VEGF and NT-proBNP levels (mean ± 95% CI) in relation to AIP degree. Following clinical relevance, degrees of invasion 3B and 3C have been clustered together based on the assumption that these AIP degrees both denote highly invasive placenta percreta, which has invaded parauterine structures such as the urinary bladder (3B) or the parametria (3C)

VEGF levels in the AIP group were significantly lower than in the control group (AIP mean 285 pg/ml, 95% CI 248–322 vs. control 391 pg/ml, 95% CI 356–426, *p* < 0.01) (Fig. 1a). NT-proBNP levels were significantly higher in the AIP group versus the control group (AIP median 329 pg/ml, IQR 287–385 vs. control 295 pg/ml, IQR 273–356, *p* = 0.03) (Fig. 1b).

### Correlation of Maternal Serum VEGF Level and AIP Degree

Maternal serum VEGF levels exhibited a significantly negative correlation with the FIGO AIP degree of invasion (Spearman’s rho = − 0.37; *p* < 0.001). This correlation remained significant even after controlling for the influence of gestational age at blood sampling through semi-partial correlation (*r* = − 0.33, *p* < 0.01). Figure 1c illustrates this correlation but shows that no linear negative correlation from normal placentation to AIP degrees of invasion 3B + 3C exists, as the median of VEGF in AIP degree of invasion 2 (placenta increta) and 3A (placenta percreta) is higher than for AIP grade 1 (placenta accreta). Grouping of grades 3B and 3C was performed due to limited case numbers in the subgroups and based on the assumption that these AIP degrees both denote highly invasive placenta percreta, which has invaded parauterine structures such as the urinary bladder or the parametria. Maternal serum NT-proBNP levels did not correlate significantly with the AIP degree (Spearman’s rho = 0.17; *p* = 0.12; Fig. 1d).

### Logistic Regression Analysis of Possible Risk Factors for AIP

The predictive power of biomarker levels and the number of previous cesarean deliveries concerning the occurrence of AIP were analyzed by univariate logistic regression analysis (Table 2). Having at least one previous cesarean delivery and levels of VEGF ≤ 328.0 pg/ml or NT-proBNP ≥ 303.5 pg/ml were detected to be significant risk factors for the occurrence of AIP. Multivariate logistic regression analysis confirmed the determining values for VEGF, NT-proBNP, and the number of previous cesarean deliveries (Table 2). Gravidity, parity, age, and the presence of placenta previa showed a significant correlation with the number of previous cesarean sections. These factors were therefore left out of the regression analysis to avoid multicollinearity.

**Table 2:** Univariate and multivariate regression analyses showing the value of variables (number of prior cesarean deliveries, maternal serum VEGF, and NT-proBNP levels) to predict AIP. Due to highly significant correlation with the number of previous cesarean sections, maternal age, gravidity, and parity are left out in regression analysis to avoid distortion due to multicollinearity. Statistically significant *p*-values (<0.05) are written in italics

	Crude OR (95% CI)	*P* value	Adjusted OR (95% CI)	*P* value
Number of prior cesarean deliveries	7.5 (3.4–16.8)	*< 0.001*	5.7 (2.5–13.1)	*< 0.001*
VEGF ≤ 328.0 pg/ml	6.3 (2.5–15.6)	*< 0.001*	3.4 (1.1–11.0)	*0.041*
NT-proBNP ≥ 303.5 pg/ml	2.9 (1.3–6.6)	*0.01*	3.7 (1.1–12.5)	*0.038*

*Abbreviations*: *VEGF* vascular endothelial growth factor, *NT-proBNP* N-terminal pro B-type natriuretic peptide, *OR (95% CI)* odds ratio (95% confidence interval)

### Predictive Value of VEGF on the Occurrence and the Outcome of AIP

**Fig. 2:**
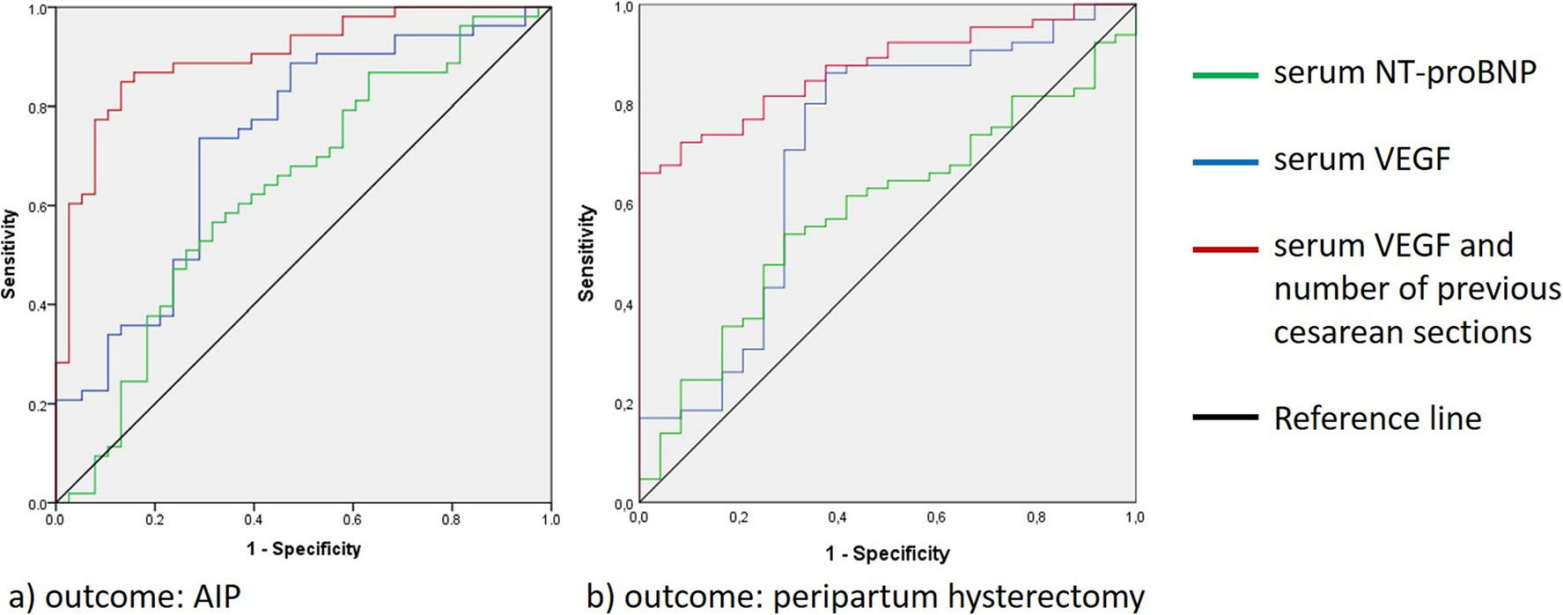
ROC curves showing the predictive value of serum VEGF and NT-proBNP in AIP. **a** Predictive value of serum biomarkers alone and in a combination of serum VEGF with the number of previous cesarean sections on the occurrence of AIP (outcome: Grading 2 to 6, VEGF: AUC = 0.729; NT-proBNP: AUC = 0.632; serum VEGF and previous cesarean deliveries: AUC = 0.915). **b** Predictive value of serum biomarkers and the need for peripartum hysterectomy (VEGF: AUC = 0.698; NT-proBNP: AUC = 0.585; serum VEGF and previous cesarean deliveries: AUC = 0.870). There was no significant difference in the hysterectomy rate between the two study centers in Liège and Berlin (*p* = 0,975, chi-squared test)

The diagnostic values of maternal serum VEGF and NT-proBNP levels in patients with AIP are summarized in Fig. 2 and Table 3. NT-proBNP level with a cut-off at 303.5 pg/ml had a negative likelihood ratio (LR−) of 0.6 and a positive likelihood ratio (LR+) of 1.6 for the outcome of AIP (AUC = 0.632, 95% CI 0.516–0.747, *p* = 0.03). A VEGF level cut-off at 328.0 pg/ml had a negative likelihood ratio of 0.4 and a positive likelihood ratio of 2.5 for the same outcome (AUC = 0.729, 95% CI 0.622–0.836, *p* < 0.001). A combination of maternal serum VEGF level and the number of prior cesarean deliveries even increased the predictive value concerning the occurrence of AIP (AUC = 0.915, 95% CI 0.853–0.977, LR− 0.1, LR+ 6.7, *p* < 0.001). The predictive value of the maternal serum VEGF level combined with previous cesarean sections was significantly better than the predictive value of the number of previous cesarean sections alone (AUC difference 0.038, *p* = 0.04). Combining VEGF and NT-proBNP levels did not yield a better predictive power than VEGF alone (AUC = 0,729, 95% CI 0,620–0,832, *p* < 0.001). Serum VEGF was able to predict the need for peripartum hysterectomy (AUC = 0.698, 95% CI 0.564–0.832, LR+ 2.4, LR− 0.3, *p* = 0.004). To show that this finding was not influenced by potentially different management in the two study centers, chi-squared test was performed and showed no significant difference in the hysterectomy rate between Liège and Berlin (*p* = 0.975).

**Table 3:** Sensitivity, specificity, negative and positive predictive value, likelihood ratios, and area under the curve (AUC) values of determined VEGF and NT-proBNP cut-off levels in patients with AIP

	Cut-off	Sensitivity	Specificity	NPV	PPV	LR−	LR+	AUC (95% CI)	*P* values
VEGF	328.0 pg/ml	73.6%	71.1%	78.9%	64.7%	0.4	2.5	0.729 (0.622–0.836)	*< 0.001*
NT-proBNP	303.5 pg/ml	65.1%	60.4%	68.5%	56.7%	0.6	1.6	0.632 (0.516–0.747)	*0.03*
Number of previous CS		91.2%	77.4%	93.2%	72.1%	0.1	4.0	0.881 (0.805–0.957)	*< 0.001*
VEGF and number of previous CS		88.2%	86.8%	92.0%	81.0%	0.1	6.7	0.915 (0.853–0.977)	*< 0.001*
NT-proBNP and number of previous CS		85.3%	83.0%	89.8%	76.2%	0.2	5.0	0.904 (0.843–0.965)	*< 0.001*

Statistically significant *p* values (< 0.05) are written in italics

*Abbreviations*: *CS* cesarean sections, *VEGF* vascular endothelial growth factor, *NT-proBNP* N-terminal pro B-type natriuretic peptide, *AUC* area under the curve, *NPV* negative predictive value, *PPV* positive predictive value, *LR−* negative likelihood ratio, *LR+* positive likelihood ratio

## Discussion

Due to its subtle ultrasound features, AIP is difficult to detect for the untrained eye. This reflects in detection rates of 50 to 66% in population studies [8–10], despite high sensitivity and specificity of ultrasound in the hands of an expert [4]. A better detection of AIP through the use of biomarkers could lead to a more convenient planning of scheduled cesarean delivery with a prepared expert team, which will result in a better outcome for the patient with less blood loss and less hysterectomies [8, 32, 33]. The study has confirmed the existence of altered maternal serum levels of both investigated angiogenic biomarkers in pregnancies affected by AIP. Serum NT-proBNP level is significantly increased in cases of AIP, while serum VEGF level was significantly reduced compared with controls, even when controlled for the number of prior cesarean deliveries per woman—the main clinical risk factor for AIP. Combining maternal serum VEGF level and the number of previous cesarean deliveries through ROC curve analysis yielded the highest predictive value on the occurrence of AIP—significantly higher than the predictive value of the number of previous cesarean sections alone. Decreased maternal serum VEGF levels significantly predicted the higher probability and need to perform a peripartum hysterectomy. This shows that serum VEGF level can be a useful part of an algorithm screening for AIP based on combined predictive values together with clinical characteristics.

Serum VEGF levels correlated inversely but not linearly with the AIP degree of invasion after controlling for gestational age at blood retrieval. Correlation analysis between serum NT-proBNP levels and the AIP degrees of invasion showed a trend but was not significant. NT-proBNP therefore seems to be of limited ability to predict AIP. VEGF appears to be the better biomarker for AIP. The question is, which pathophysiological process of AIP leads to the observed alterations in maternal serum VEGF and NT-proBNP levels? It is known that placental invasiveness is promoted by neoangiogenesis [34, 35]. Subplacental and uterovesical hypervascularity in AIP presents both in antenatal sonography [29] and in postpartum histological sections of placental beds [36]. Therefore, we hypothesized that NT-proBNP and VEGF as vasculogenic factors are elevated in maternal serum. Interestingly, in accordance with the study by Wehrum et al., who collected serum samples at an earlier mean gestational age of 31 (28–34) weeks prior to steroid administration and blood transfusion, serum VEGF levels appeared to be lower in our AIP cohort [21]. This finding could be explained by an underlying pathophysiological mechanism in AIP: the role of oxidative stress. Placental hypoxia—and therefore reduced oxidative stress—stimulates increased placental invasiveness by enhancing the expression of VEGF [37]. According to the currently accepted hypothesis of AIP pathogenesis, AIP mainly develops when nidation has taken place in the region of a uterine scar—a region of localized hypoxia due to abnormal vascularization that has resulted from the scarring process after surgery [21, 38–40]. We presume that VEGF levels might be elevated during the first and second trimester, which would explain increased neoangiogenesis in AIP. When normoxia—or even hyperoxia—has been achieved through increased neoangiogenesis in AIP, augmented oxidative stress might lead to a downregulation of VEGF [41, 42]. As blood was sampled during the third trimester (median GA 35 weeks, IQR 35–36 weeks), this hypothesis could explain the paradoxically low serum VEGF levels in cases of AIP.

The strength of the study lies in the size of the cohort. Especially the comparatively large number of cases of placenta percreta (degrees of invasion 3A–3C: *n* = 32) stands out against the sizes of the existing studies. Still no differentiation in VEGF levels between clinical AIP degrees (degrees of invasion 1—3C according to FIGO [3]) could be seen. This limitation shows that for further evaluation of reasonable VEGF thresholds, an even larger number of patients are needed. Although the studied patient collective of *n* = 44 is large against the background of the rarity of AIP, the number of patients in the subgroups (AIP degrees of invasion 1–3C) is still rather small. For example, only three patients with AIP degree 3C (placental infiltration of the parametrium) were recruited in the study. Therefore, the statistical power of the subgroup analysis is limited.

Various physiological situations can alter human biomarker levels. Serum NT-proBNP levels can be increased by physiological stress and cardiac strain [43–45]. We controlled for this confounding factors by retrieving blood pre-operatively. VEGF can be altered by a person’s body mass index (BMI). The individuals in the AIP group did not have significantly different BMIs compared with the control group (Table 1). A slight increase in serum VEGF levels during the course of pregnancy has been shown in other studies [46–48]. To control for this effect (which is much stronger in preeclamptic pregnancies), the groups were matched for gestational age at blood retrieval. Placenta previa does not seem to influence maternal serum levels of VEGF and NT-proBNP [21, 22, 24].

We hypothesized that biomarker levels might be altered most significantly later in pregnancy, when the process of placentation is far advanced. As most of the blood samples were drawn during third trimester, the usefulness of determining these biomarkers earlier in pregnancy—which would be the prerequisite for a useful biomarker—still must be investigated. Two studies exist (*N* = 48; *N* = 70), which have documented maternal serum VEGF at 3 or 5 time points during pregnancy in women with preeclampsia, gestational hypertension, or normotension [47, 48]. Due to this limited evidence, no normal values or standard curves according to gestational age are available for VEGF and the measured biomarker levels cannot yet be expressed as multiples of the median. However, VEGF levels appear to be very stable throughout normotensive pregnancies [47, 48]. However, the study shows that the biomarker levels are still altered in third trimester. This indicates that VEGF could also aid birth clinics in determining their management for patients who are referred late in pregnancy.

The degree of biomarker levels depends on the specific immunoassay used. For VEGF, different research groups have recorded different levels in pregnancy: 0.02–11 pg/ml (R&D Systems, Minneapolis, USA) [21], 3–9 pg/ml (Cat No: SEA143Hu, Lot No: L150303051 USCN life science Inc., Wuhan, Hubei, China) [24], and 35–110 pg/ml (Cat No: E-EL-H2569, Elabscience, Wuhan, Hubei, China) [23]. Palm et al. could only detect VEGF (above 9 pg/ml) in 15% of normal pregnant women beyond 33 weeks (Cat No: DVE00, R&D Systems, Minneapolis, MN, USA) [46]. In this study, women had VEGF levels of 240–430 pg/ml. For NT-proBNP, the most favorable cut-off level to differentiate between AIP and normal placentation as determined by ROC curve analysis in this study was 303.5 pg/ml. Ersoy et al. proposed a cut-off at 125.85 pg/ml (manufacturer of assay not stated in the publication) [22]. Standard curves for NT-proBNP according to gestational age are also not available. Only Franz et al. calculated multiples of the meridian using an electrochemiluminescence immunoassay (*N* = 94) [49]. Before general clinical implementation, more studies are needed to define concrete cut-off values for reliable prediction of AIP (and its degrees of invasion). Biomarkers cannot replace a detailed ultrasound scan. However, in comparison with the aforementioned AIP detection rates of 50 to 66%, a routine assessment of the number of previous cesarean sections and VEGF level could lead to a relative risk reduction of 60 to 70% for the event of an unsuspected AIP [8–10].

## Conclusion

Third trimester levels of VEGF, more than NT-proBNP, can help in predicting AIP and the need for hysterectomy prior to delivery—especially in combination with clinical factors such as the number of prior cesarean deliveries. Biomarker levels did not allow a clear antenatal classification of the degree of placental invasion. A maternal serum biomarker hinting at AIP can be helpful in outpatient settings where no specialized ultrasound scan is available to help to identify patients at high risk of AIP and refer them to a specialized center for further diagnostics. As a next step, we propose to determine maternal serum VEGF levels in pregnancies complicated by AIP starting from first trimester onwards.

## Data Availability

All raw data are available upon request.
